# Building a better neonatal mouse model to understand infant respiratory syncytial virus disease

**DOI:** 10.1186/s12931-015-0244-0

**Published:** 2015-08-01

**Authors:** Dahui You, David T. Siefker, Bishwas Shrestha, Jordy Saravia, Stephania A. Cormier

**Affiliations:** Department of Pediatrics, University of Tennessee Health Science Center, Memphis, TN USA; Children’s Foundation Research Institute, Le Bonheur Children’s Hospital, Memphis, TN USA

**Keywords:** RSV, Neonatal mouse model, rA2-19F

## Abstract

**Background:**

Respiratory syncytial virus (RSV) is the number one cause of lower respiratory tract infection in infants; and severe RSV infection in infants is associated with asthma development. Today, there are still no vaccines or specific antiviral therapies against RSV. The mechanisms of RSV pathogenesis in infants remain elusive. This is partly due to the fact that the largely-used mouse model is semi-permissive for RSV. The present study sought to determine if a better neonatal mouse model of RSV infection could be obtained using a chimeric virus in which the F protein of A2 strain was replaced with the F protein from the line 19 clinical isolate (rA2-19F).

**Methods:**

Five-day-old pups were infected with the standard laboratory strain A2 or rA2-19F and various immunological and pathophysiological parameters were measured at different time points post infection, including lung histology, bronchoalveolar lavage fluid (BALF) cellularity and cytokines, pulmonary T cell profile, and lung viral load. A cohort of infected neonates were allowed to mature to adulthood and reinfected. Pulmonary function, BALF cellularity and cytokines, and T cell profiles were measured at 6 days post reinfection.

**Results:**

The rA2-19F strain in neonatal mice caused substantial lung pathology including interstitial inflammation and airway mucus production, while A2 caused minimal inflammation and mucus production. Pulmonary inflammation was characterized by enhanced Th2 and reduced Th1 and effector CD8^+^ T cells compared to A2. As with primary infection, reinfection with rA2-19F induced similar but exaggerated Th2 and reduced Th1 and effector CD8^+^ T cell responses. These immune responses were associated with increased airway hyperreactivity, mucus hyperproduction and eosinophilia that was greater than that observed with A2 reinfection. Pulmonary viral load during primary infection was higher with rA2-19F than A2.

**Conclusions:**

Therefore, rA2-19F caused enhanced lung pathology and Th2 and reduced effector CD8^+^ T cell responses compared to A2 during initial infection in neonatal mice and these responses were exacerbated upon reinfection. The exact mechanism is unknown but appears to be associated with increased pulmonary viral load in rA2-19F vs. A2 infected neonatal lungs. The rA2-19F strain represents a better neonatal mouse model of RSV infection.

**Electronic supplementary material:**

The online version of this article (doi:10.1186/s12931-015-0244-0) contains supplementary material, which is available to authorized users.

## Background

Respiratory Syncytial Virus (RSV) is the number one cause of lower respiratory tract infection [1]. RSV is also among the most important pathogens responsible for childhood pneumonia [2]. It has been estimated that 2.1 million children in the United States alone require medical attention for RSV infection each year [3].

There is still no vaccine or specific anti-viral therapeutics available. Currently, only a humanized monoclonal antibody against the fusion (F) protein of RSV, Palivizumab, is approved for use for high-risk infants [4], and the antiviral Ribavirin, which has been used, is not specific for RSV and its efficacy is controversial [5]. An early attempt at developing a formalin-inactivated RSV vaccine failed tragically in the 1960s [6, 7]. The failure of this RSV vaccine highlighted our lack of knowledge of the interaction of RSV with the infant immune system.

Many animal models of RSV infection have been used to study the mechanisms of RSV pathogenesis in human infants [8]. Mouse models are particularly useful because of the many genetically modified strains and an abundance of molecular biology tools and reagents commercially available. Our laboratory and others have used neonatal mouse models of infection to more closely mimic the interaction between RSV and the human infant immune system [9–11]. It has been found that neonatal mice (i.e., <7 days of age) represent a more age-appropriate model to study RSV immunopathology. For example, neonatal mice mount a T-helper type 2 (Th2) response to RSV infection as do human infants [10]. Secondary infection of mice initially infected as neonates resulted in airway inflammation, mucus production, and breathing difficulty, all symptoms of human RSV disease [11]. Importantly, our laboratory has shown that mice infected with high doses of RSV as neonates are predisposed to develop pulmonary complications later in life, as is observed in humans [9, 12]. However, the current mouse models of RSV infection still fall short of reproducing some of the pathologies seen in human infants such as interstitial pneumonitis and alveolitis [13], which is believed to be due to the fact that mice are only semi-susceptible to human RSV infection [14].

The need for a better mouse model of RSV infection has also led to identification of RSV strains that induce symptoms in mice more consistent with those of humans. The Long and A2 strains (originally isolated from clinical samples in 1957 and 1961, respectively) have long been the standard laboratory RSV strains used in research [15, 16]. However, these historical strains induce less pathology in adult mice compared to more recently isolated strains, including the Line 19 strain [17, 18]. Fusion (F) protein diversity has been shown to be an important factor responsible for pulmonary disease severity in adult mice [17]. Recently, a chimeric RSV in which the F protein from A2 strain was replaced with the F protein from the Line 19 strain (rA2-19F) was produced [17]. Infection of adult mice with rA2-19F resulted in higher pulmonary viral loads compared to either of its parent strains (A2 or line 19) [17]. In an effort to generate a better neonatal mouse model of RSV infection, we show here that neonatal mice infected with the chimeric rA2-19F strain of RSV, compared to the standard A2 strain, exhibited enhanced lung pathology, including interstitial pneumonitis and alveolitis, enhanced Th2 responses, and reduced effector CD8^+^ T cells during both primary and secondary infections. The enhanced pathology and aberrant immune responses correlated with higher pulmonary viral load in the neonatal lungs during primary infection.

## Materials and methods

### Mice

BALB/c breeders were purchased from Harlan Laboratories (Indianapolis, IN, USA) and bred in specific-pathogen-free facilities at University of Tennessee Health Science Center (UTHSC, Memphis, TN, USA). Pups born on the same date were used for experiments.

All animal experiments were performed according to the Guide for the Care and Use of Laboratory Animals and approved by the Institutional Animal Care and Use Committee at UTHSC.

### Viruses and the infection

Human RSV A2 strain was purchased from Advanced Biotechnologies Inc (Columbia, MD, USA) and passaged in Vero cells (ATCC; Manassas, VA, USA) cultured in serum-free-media (SFM4MegaVir; Hyclone, Logan, UT, USA). The chimeric strain rA2-19F was a gift from Dr. Martin Moore (University of Emory, Atlanta, GA, USA) [17] and passaged in our laboratory as RSV A2 above.

Mice were infected intranasally with RSV at a dose of 10^4.68^ TCID_50_ (tissue culture infectious dose) per gram of body weight. Sham mice were inoculated with culture media collected in the same way as virus stocks.

### Pulmonary viral load

At various time points after primary RSV infection, lungs were isolated and stored at −80 °C until viral load analysis by either RT-qPCR [19] or TCID_50_ [20, 21] methods. For real time qPCR, total RNA was isolated from lungs using Qiagen RNeasy plus mini kit (Valencia, CA, USA); and one step RT-qPCR was performed using SuperScript® III Platinum®One-Step qRT-PCR Kit from Life Technologies (Carlsbad, CA, USA). Relative expression of the virus NS1 gene in lungs was normalized to Hprt using ∆∆Ct method and the primers used were:

NS1, forward: 5′-CACAACAATGCCAGTGCTACAA-3′

NS1, reverse: 5′-TTAGACCATTAGGTTGAGAGCAATGT-3′

Hprt, forward: 5′-GGCTCCGTTATGGCGACCCG-3′

Hprt, reverse: 5′-CGAGCAAGACGTTCAGTCCTGTCC-3′

For TCID_50_ assay, lungs were homogenized and supernatants were used to infect Vero cells on a 96-well plate. After incubation in 37 °C at 5%CO_2_ for 7 days, the wells showing cytopathic effect were counted and TCID_50_ calculated as per the Spearman-Kärber method [20, 21].

### Bronchoalveolar lavage fluid cellularity

Bronchoalveolar lavage fluid (BALF) was isolated in 0.25 ml (neonates) or 1 ml (adults) of PBS containing 0.5 % BSA. BALF cells were enumerated, spun onto slides, and stained with Hema-3 staining kit (Thermo Fisher Scientific, Waltham, MA, USA). Two unbiased observers differentiated the cellularity using standard morphological criteria. Supernatants were collected and stored at −80 °C till cytokine analysis.

### Pulmonary T cell profile

Lung single cells were prepared using gentleMACS™ Octo Dissociator (Miltenyi Biotec, San Diego, CA, USA). After red blood cell lysis, the cells were stimulated for 5 h with 5 ng/ml phorbol-12-myristate-13-acetate (PMA) and 500 ng/ml ionomycin (Sigma-Aldrich, St. Louis, MO) in the presence of GolgiPlug (1 μl/10^6^ cells; BD Biosciences, Franklin Lakes, NJ). After stimulation, the cells were stained with fixable viability dye and antibodies to CD3 (17A2), CD4 (RM4-4; Biolegend, San Diego, CA, USA), CD8 (53–6.7), IFNγ (XMG1.2), and IL4 (BVD6-24G2). Stained cells were analyzed on a Canto II flow cytometer (BD Biosciences) and plotted with FlowJo software (v10 for Windows; Tree Star; Ashland, OR, USA). All antibodies and viability dye were from eBioscience (San Diego, CA, USA) unless otherwise stated.

### Lung histopathology

Lungs were inflated at a constant fluid pressure of 25 cm and fixed with zinc formalin (Thermo Fisher Scientific). After fixation, lungs were embedded and sections cut and stained by the pathology core at UTHSC. Hematoxylin and eosin (H&E) were used to identify cellular infiltrates and periodic acid-Schiff (PAS) for mucus in the airway epithelial cells.

### Pulmonary function

Airway resistance to methacholine (MeCh; Sigma-Aldrich) was measured using the FlexiVent FX system (Scireq, Montreal, QC, Canada). Raw data were collected by fitting into a single –compartment model and normalized by subtracting individual baseline values at 0 mg/ml MeCh.

### Cytokine levels in the airways or the lung

The kinetics of IL13 expression in the lung after primary infection was measured using real-time qPCR. Total lung RNA was isolated using Qiagen RNeasy plus mini kit and reverse-transcribed to cDNA using SuperScript III first-strand synthesis system. Real-time PCR was then performed using Power Sybr green PCR master mix (Life Technologies) and the relative expression of IL13 were normalized to Hprt using ∆∆Ct method. The primers for IL13 were as follows and the primers for Hprt were as above.

Forward primer: 5′-TGATGAGCTACTACTGGTCAGC-3′

Reverse primer: 5′-GATCTCTTAGCACAAGGATGGC-3′

The level of other cytokines in the BALF including IFNγ, IL12 (p40), and IL4 were measured using Milliplex mouse cytokine/chemokine assay kit (Millipore Corporation; Billerica, MA, USA) on Luminex 200 system (Luminex, Austin, TX, USA). IL13 level in the BALF were measured by ELISA kit from eBioscience following manufacturer’s instructions. Data below detection limit of each cytokine were excluded.

### Statistical analysis

Data were plotted as means ± standard errors (SEM) by Prism 6 (GraphPad Software; La Jolla, CA, USA). Pulmonary function test results were analyzed using two-way analysis of variance and Bonferroni post-hoc tests. All other data were analyzed using Student’s *t*-test (2 groups) or multiple *t* test with Holm-Sidak correction (3 groups). Differences were considered significant if *p* < 0.05. Every experiment was repeated at least twice.

## Results

### Primary rA2-19F infection induced higher pulmonary viral loads than A2 and line 19

**Fig. 1:**
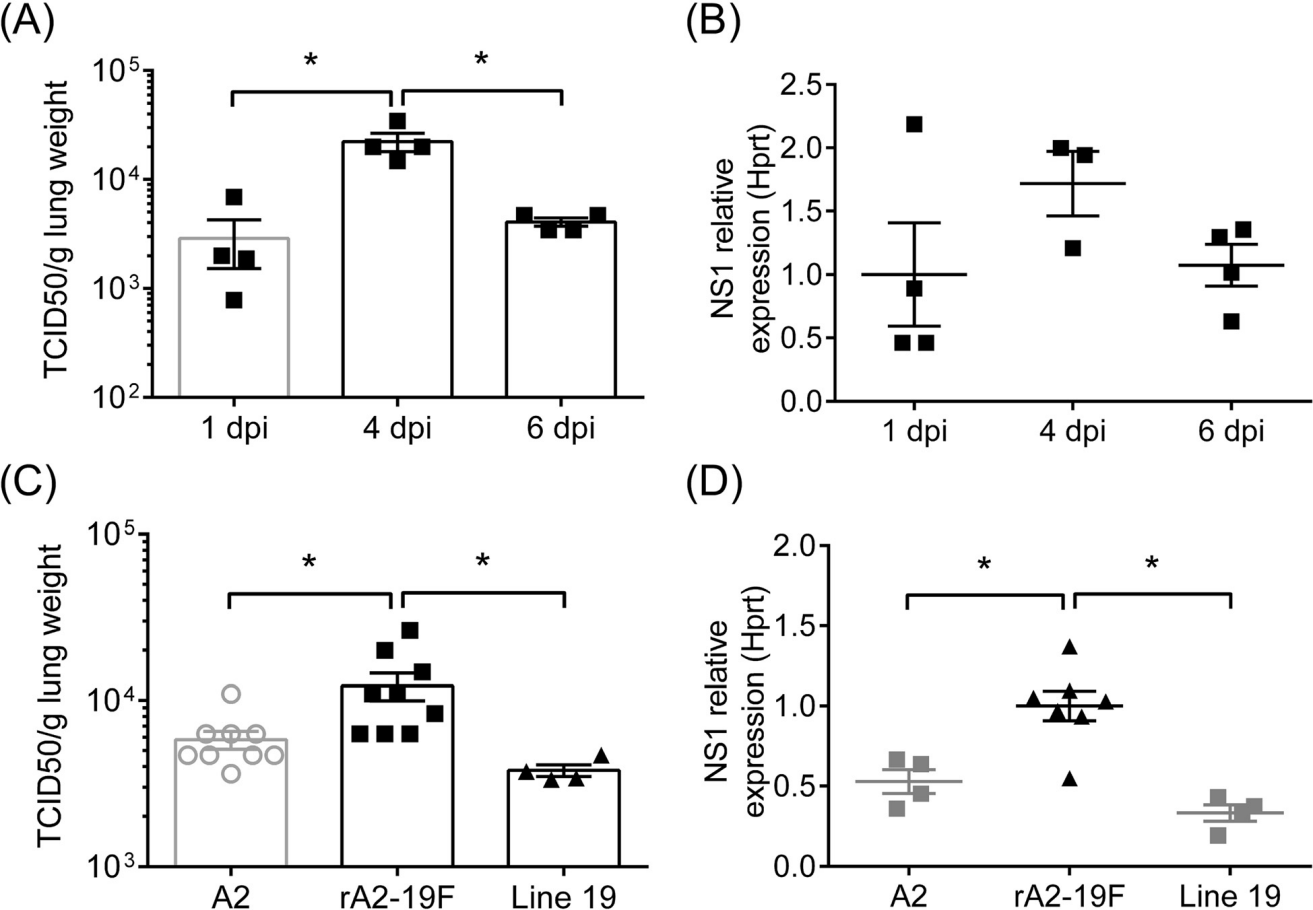
Pulmonary viral loads were elevated in the lungs of neonatal mice infected with rA2-19F. Lungs were isolated and viral load determined at various time points. **a** Viral load kinetics of rA2-19F infection determined by TCID_50_ method. *N* = 4. **b** Viral load kinetics of rA2-19F infection determined by qPCR (relative expression of RSV NS1 gene). *N* = 3-4. **c** Viral load by TCID_50_ assay at 4 dpi. *N* = 4-9. **d** Viral load by qPCR at 4 dpi. *N* = 4-7. A2: Neonates infected with A2 strain; rA2-19F: Neonates infected with rA2-19F. Line 19: Neonates infected with line 19. These figures are representative of 2 independent experiments. *:*p* < 0.05

Human infant data demonstrate a positive correlation between RSV viral load and disease severity [22, 23]; and infection of adult mice with rA2-19F resulted in higher pulmonary viral loads compared to either of its parent strains (A2 or line 19) in the lung [17]. We therefore tested if this correlation stayed true in neonatal mice. To test this, pups were infected with rA2-19F, and pulmonary viral load was measured at various time points after infection by either real-time PCR (Fig. 1a) or TCID_50_ assay (Fig. 1b). This confirmed that peak viral load occurred at 4 days post-infection (dpi) in neonatal mice infected with rA2-19F, as in neonates infected with A2 [24] or line 19 [25]. We then compared the peak viral load at 4 dpi in neonatal mice infected with A2, line 19, or rA2-19F. TCID_50_ assay shows that more live viruses were present in rA2-19F than A2 or line 19 infected lungs (Fig. 1c; 12,247 ± 2,321 vs. 5,804 ± 719 or 3,795 ± 309 TCID_50_/g lung, respectively). Real-time PCR confirms that the relative expression of the virus NS1 gene was significantly higher in rA2-19F compared to A2 or line 19 infected lungs (Fig. 1d; 1.00 ± 0.09 vs. 0.53 ± 0.07 or 0.33 ± 0.05, respectively). No differences in pulmonary viral loads were observed between A2 and line 19 infected neonates. Since our hypothesis was that an RSV strain demonstrating higher viral load would more closely mimic human disease and since human infant data demonstrate a positive correlation between RSV viral load and disease severity, line 19 was not included in the rest of the study.

### Primary rA2-19F infection caused more lung pathology than A2 in neonates

**Fig. 2:**
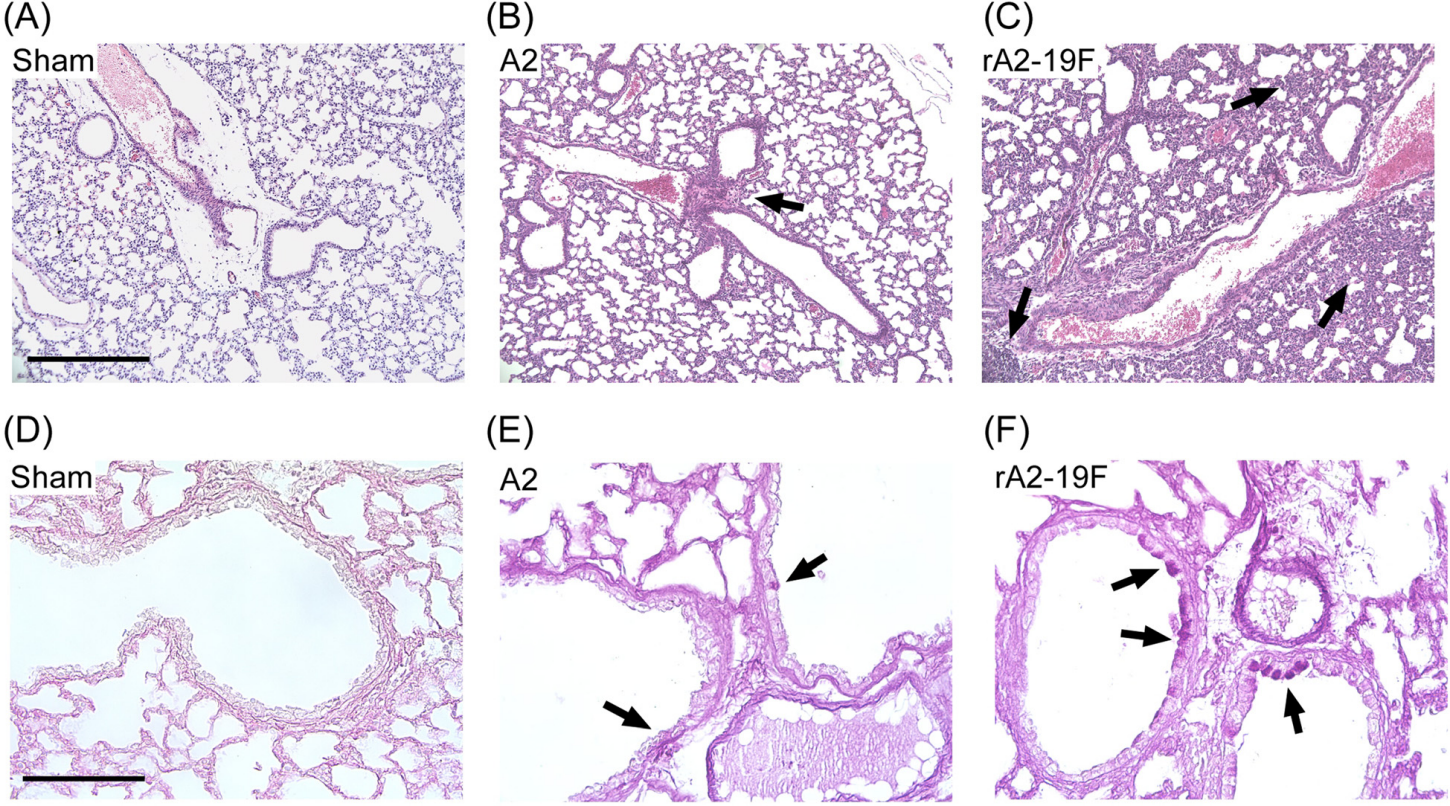
Neonatal infection with rA2-19F induced significant lung pathology. Five-day old pups were infected with media, rA2-19F or A2, and lungs were isolated, fixed, and stained. **a**–**c** H&E staining for inflammation at 6 dpi. Scale bar = 400um. Arrows indicate inflammation. **d**–**e** PAS staining for airway mucus production at 8 dpi. Scale bar = 100 um. Arrows indicate mucus producing cells. Sham: Neonates infected with media; A2: Neonates infected with A2 strain; rA2-19F: Neonates infected with rA2-19F. These pictures are representative of 2 independent experiments; 3 mice per experiment time point

Lung pathology is the most direct method to measure severity of infection; therefore, we performed histological examination on the lungs isolated from pups infected with media (Sham), rA2-19F, or A2. As shown in Fig. 2, five-day-old pups infected with rA2-19F exhibited substantial peribronchiolar and interstitial inflammation; whereas pups infected with A2 only showed mild inflammation at 6 dpi (Fig. 2b & c). Infection with rA2-19F also induced mucus production in airway epithelial cells; whereas infection with A2 induced sporadic amounts of mucus in airway cells at 8 dpi (Fig. 2e & f). Sham mice showed no signs of inflammation or mucus production (Fig. 2a & d).

### Primary rA2-19F infection induced aberrant immune responses in the neonatal lung

**Fig. 3:**
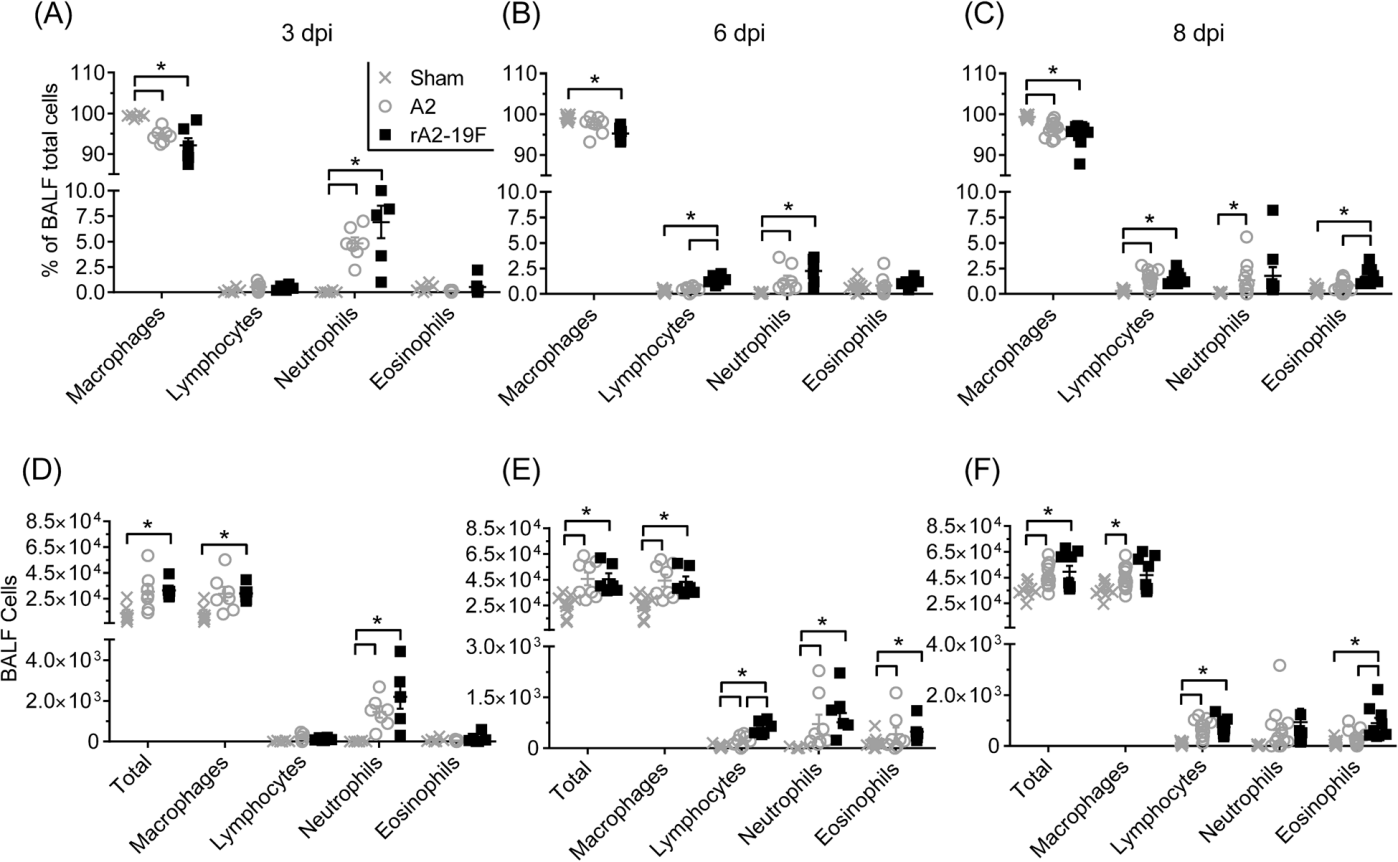
Neonatal infection with rA2-19F increased airway inflammation compared to infection with the A2 strain. Pups were infected with media, rA2-19F or A2, and BALF was isolated and cells enumerated at 3, 6, and 8 dpi. **a**–**c** BALF cellularity expressed as a percentage of total cells. *N* = 5-13. **d**–**f** BALF cellularity expressed as cell numbers. *N* = 5-13. Sham: Neonates infected with media; A2: Neonates infected with A2 strain; rA2-19F: Neonates infected with rA2-19F. The figure is a representative of 3 independent experiments. *:*p* < 0.05

Immunopathologies (i.e., Th2 biased immune responses) have been indicated in RSV-induced disease in both human [7] and animal studies [26]. We sought to determine if rA2-19F infection recapitulated the typical immunopathologies associated with RSV in neonatal mice. To do this, pups were infected with either rA2-19F or A2, and the pulmonary immune responses were determined at various time points post-infection. Both RSV infections induced macrophages, neutrophils, lymphocytes, and eosinophils into the bronchoalveolar lavage fluid (BALF; Fig. 3d –f: Sham vs. A2 or rA2-19F). We observed an increase in the frequency of lymphocytes at 6 dpi (Fig. 3b & c; 1.37 ± 0.19 vs. 0.45 ± 0.91 %) and eosinophils at 8 dpi (1.71 ± 0.29 vs. 1.34 ± 0.44 %) in neonates infected with rA2-19F compared to A2. The numbers of lymphocytes (Fig. 3e & f; 605 ± 82 vs. 224 ± 55 cells/ml) and eosinophils (898 ± 211 vs. 317 ± 83 cells/ml) were consistently increased. No differences were observed between the two groups at 3 dpi in either cell frequencies (Fig. 3a) or numbers (Fig. 3d).

**Fig. 4:**
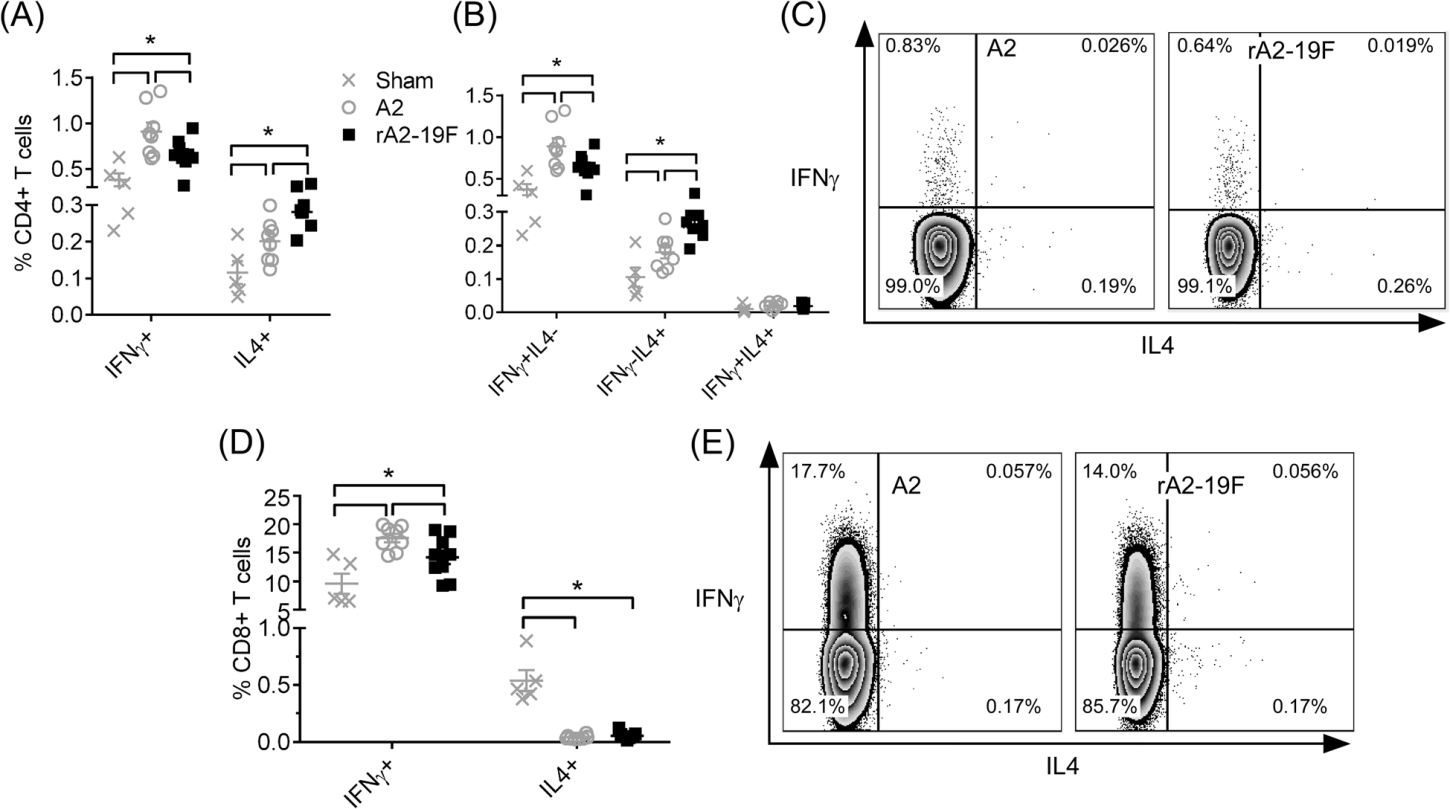
Neonatal infection with rA2-19F exacerbated Th2 and reduced effector CD8^+^ T cell responses in lungs. Pups were infected with media, rA2-19F or A2, and single cells were isolated from lungs and stained with CD3, CD4, CD8, IFNγ, and IL4 for flow cytometry at 6 dpi. **a** CD4^+^ T cells expressing IFNγ or IL4. *N* = 5-9. **b** CD4^+^ T cell subsets (Th1: IFNγ + IL4-; Th2: IFNγ-IL4+; multi-functional Th: IFNγ + IL4+). *N* = 5-9 (**c**) Representative flow plot of (**a**). **d** CD8^+^ T cell expressing IFNγ or IL4. *N* = 5-9. **e** Representative flow plot of (**d**). Sham: Neonates infected with media; A2: Neonates infected with A2 strain; rA2-19F: Neonates infected with rA2-19F. These figures are representative of 2 independent experiments. *:*p* < 0.05

To further delineate the T cell populations in the lung, we measured the pulmonary T cell profile at 6 dpi using flow cytometry (Fig. 4; gating strategy refers to Additional file 1: Figure S1). Primary RSV infection of neonates induced both Th1 and Th2 cells in the lung (Sham vs. A2 or rA2-19F). However, the frequency of IL4 expressing CD4^+^ T cells (0.28 ± 0.01 vs. 0.20 ± 0.02 %) cells was greater and the frequency of IFNγ expressing CD4^+^ T cells (0.66 ± 0.06 vs. 0.91 ± 0.10 %) was lower in the lungs of rA2-19F vs. A2 infected mice (Fig. 4a). The IL4 expressing CD4^+^ T cells appear to be Th2 cells (CD4 ^+^ IFNγ^-^IL4^+^), while the IFNγ expressing CD4^+^ T cells appear to be mainly Th1 cells (CD4^+^ IFNγ ^+^ IL4^-^) (Fig. 4b,c). Few multi-functional T cells (CD4^+^ IFNγ ^+^ IL4^+^ and CD8^+^ IFNγ ^+^ IL4^+^) were found in the neonatal lung (Fig. 4b, c, d, e). Additionally, a reduced frequency of effector CD8^+^ T cells (CD8^+^ IFNγ^+^) were found in rA2-19F compared to A2 infected lungs (14.18 ± 1.20 vs. 17.56 ± 0.75 %) (Fig. 4 d & e).

### Primary rA2-19F infection induced more pulmonary IL13 expression than A2 in neonates

**Fig. 5:**
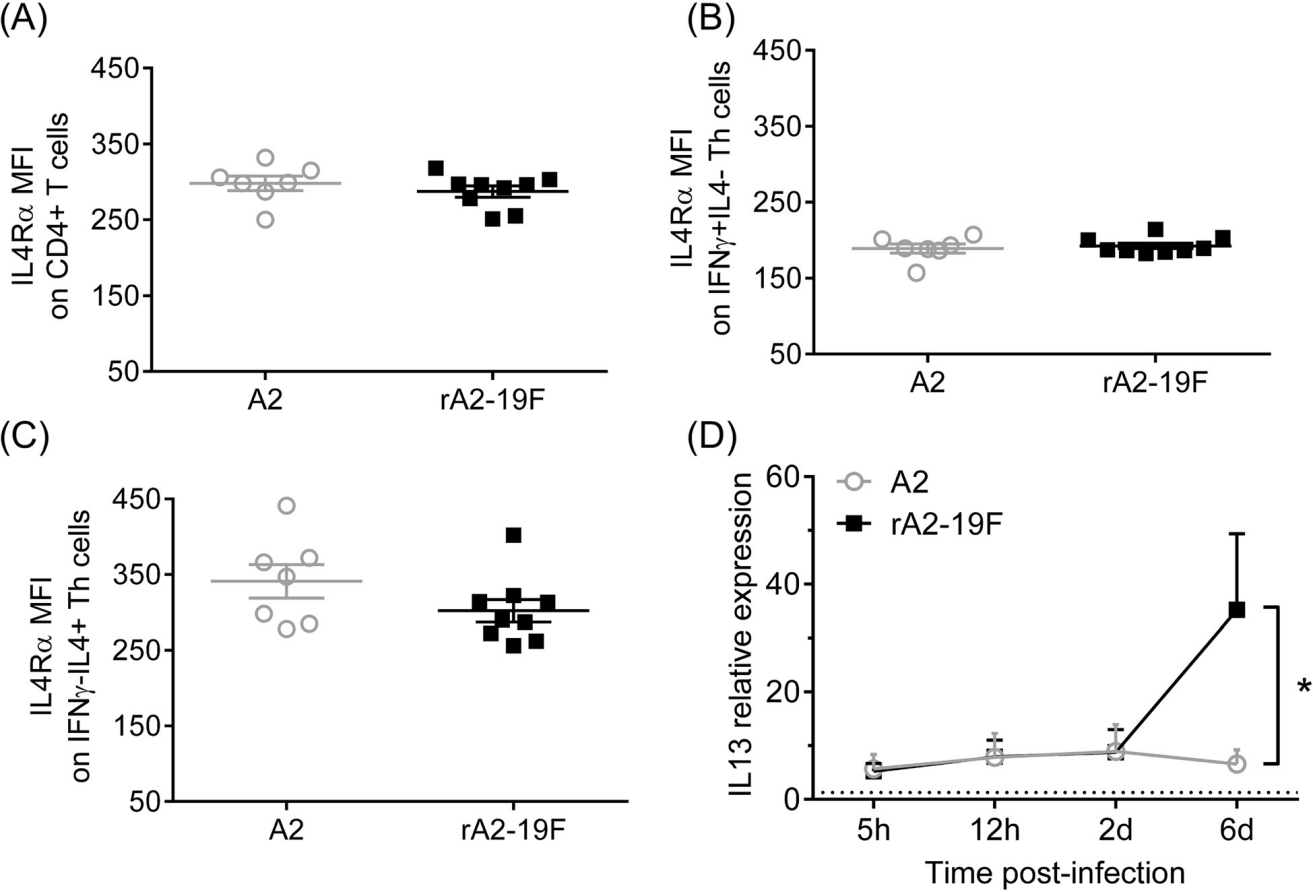
rA2-19F induced more pulmonary IL13 expression during primary infection. **a**–**c** Relative expression of IL4Rα on T cell subsets. Pups were infected with rA2-19F or A2, and single cells were isolated from lungs and stained with IL4Rα, CD3, CD4, IFNγ, and IL4 for flow cytometry at 6 dpi. MFI: mean fluorescence intensity. *N* = 7-9. **d** Relative expression of IL13 in the lungs. Pups were infected with rA2-19F, A2, or mock, and RNA from total lung homogenates was isolated at 5 h, 12 h, 2 days, and 6 days. The relative expression of IL13 in the infected lungs was determined using qPCR and normalized to corresponding mock infected lungs. *N* = 6-9. A2: Neonates infected with A2 strain; rA2-19F: Neonates infected with rA2-19F. These figures are representative of 2 independent experiments. *:*p* < 0.05

Both human [27, 28] and animal model [26] studies have demonstrated that Th2 biased immune responses during RSV infection play a pathogenic role. Our lab has published several reports [29, 30] showing that pulmonary T cells (and other cells) in neonatal mice express elevated levels of IL4Rα, a component of the receptor for both IL4 and IL13, than adult mice; and the downregulation of pulmonary IL4Rα expression or deletion of IL4Rα specifically on CD4^+^ T cells substantially ablated the pathophysiologies associated with both primary and secondary RSV infection in mice initially infected as neonates. We hypothesized that rA2-19F might exacerbate lung pathologies via the same mechanism (i.e., elevating IL4Rα levels or signaling). Therefore, we measured the expression of IL4Rα (receptor) on CD4^+^ T cells by flow cytometry and IL13 (ligand) expression in the lung by real-time PCR during primary infection. No significant differences were detected in IL4Rα expression on CD4^+^ T cells or its subtypes between rA2-19F and A2 infected lungs (6 dpi, Fig. 5a–c). However, IL13 expression was significantly increased in rA2-19F infected mouse lungs compared to A2 infected lungs at 6 dpi (Fig. 5d; 35.22 ± 14.14 vs. 6.58 ± 2.64). This increase in IL13 expression at 6 dpi is consistent with the observation that Th2 cells were increased at this same time point in rA2-19F compared to A2 infected mice (Fig. 4).

### Lung pathology was exacerbated following secondary infection with rA2-19F

The failed vaccine trial in the 1960s showed that RSV-FI vaccinated infants developed more severe lung disease than the placebo during natural RSV infection [7]. Therefore, we and others [10, 24, 31] utilize an RSV reinfection model to model this phenomenon. This model has effectively demonstrated that RSV infects and replicates in the lung [11] and induces substantial pulmonary pathophysiologies following reinfection [10, 24, 31].

**Fig. 6:**
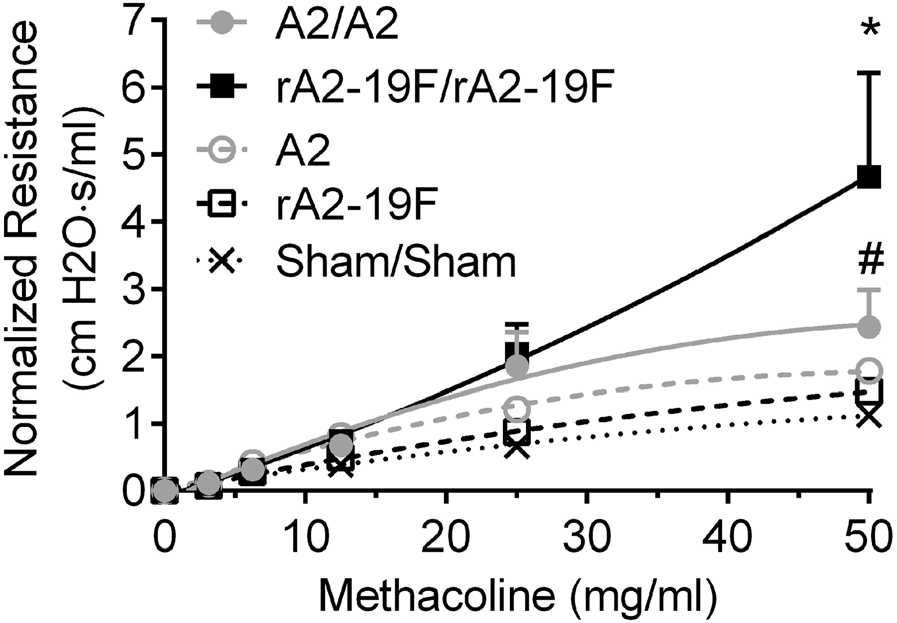
Reinfection of mice with rA2-19F enhanced airway hyperreactivity. Pups were infected and reinfected as young adults with media, rA2-19F or A2 at 4 weeks post primary infection. Six days post reinfection, lung resistance was measured using FlexiVent. N = 5-7. Sham/Sham: mice initially infected with media as neonates and reinfected with media as young adults; A2: mice only infected with A2 as neonates; rA2-19F: mice only infected with rA2-19F as neonates; A2/A2: mice initially infected with A2 as neonates and reinfected with A2 as young adults; rA2-19F/rA2-19F: mice initially infected with rA2-19F as neonates and reinfected with rA2-19F as young adults. The figure is a representative of 2–3 independent experiments. *:*p* < 0.05

To determine if rA2-19F further exacerbated lung pathologies during reinfection, pups were infected with either rA2-19F or A2 and reinfected with the same virus strain at 4 weeks post-primary infection. As illustrated in Fig. 6, both A2 and rA2-19F reinfected mice showed greater airway resistance than sham infected or single infected mice. However, airway resistance in rA2-19F reinfected mice was significantly greater than A2 reinfected mice (4.67 ± 1.54 vs. 2.44 ± 0.56 cm H2O∙s/ml).

**Fig. 7:**
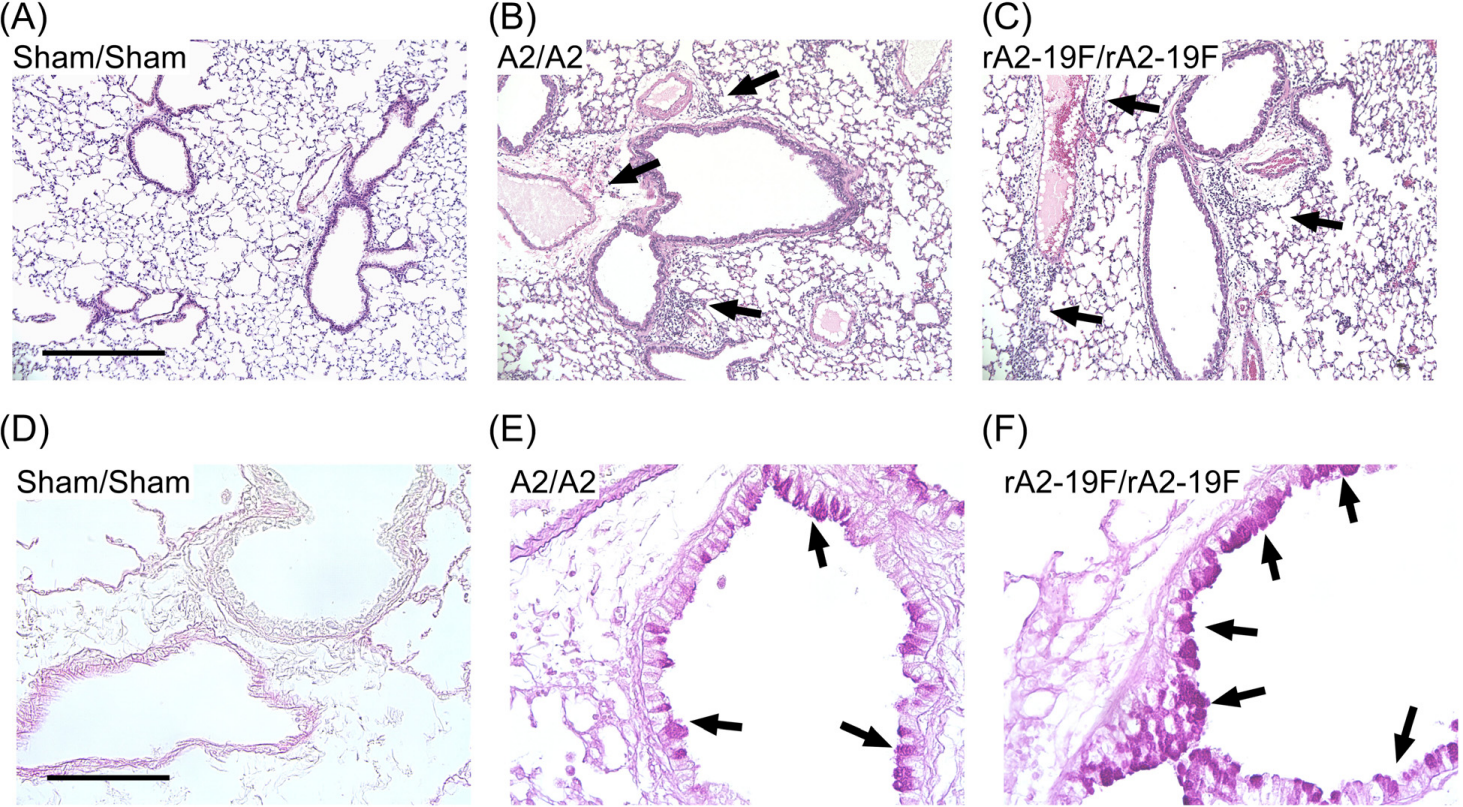
Reinfection of rA2-19F worsened lung pathology. Pups were infected and reinfected as young adults with media, rA2-19F or A2. Six days post reinfection, lungs were isolated, fixed, and stained for histopathology. **a**–**c** H&E staining for inflammation. Scale bar = 100um. Arrows indicate inflammatory cell aggregates. **d**–**f** PAS staining for airway mucus production. Scale bar =400 um. Arrows indicate mucus producing cells. Sham/Sham: mice initially infected with media as neonates and reinfected with media as young adults; A2/A2: mice initially infected with A2 as neonates and reinfected with A2 as young adults; rA2-19F/rA2-19F: mice initially infected with rA2-19F as neonates and reinfected with rA2-19F as young adults. The figure is a representative of 2 independent experiments; 3 mice per experiment time point

Histological examination of the lungs confirmed that overall pulmonary inflammation appeared similar between the two groups (Fig. 7b & c); whereas rA2-19F reinfection induced greater airway mucus production compared to A2 reinfected lungs (Fig. 7e & f). No inflammation and mucus production was obvious in sham infected mice (Fig. 7a & d).

### Secondary rA2-19F infection exacerbated the aberrant immune responses

**Fig. 8:**
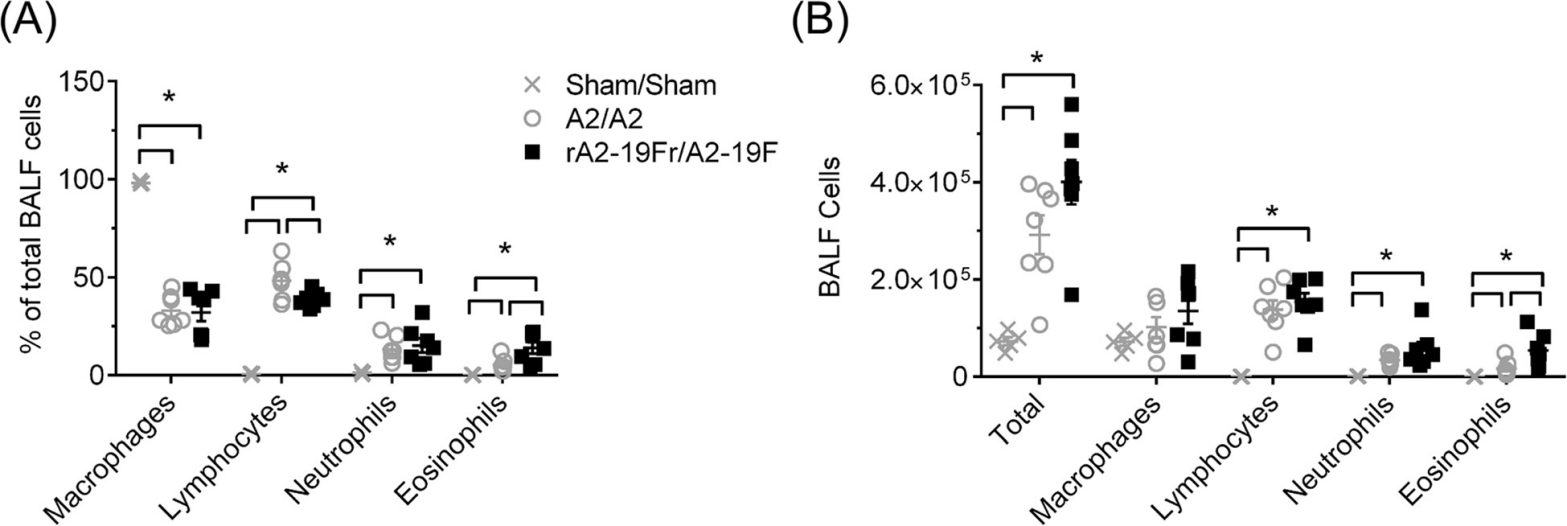
Airway inflammation is increased following reinfection with rA2-19F. Pups were infected and reinfected as young adults with media, rA2-19F or A2. Six days post reinfection, BALF was isolated and enumerated. **a** BALF cellularity expressed as a percentage. *N* = 7. **b** BALF cellularity expressed as cell numbers. *N* = 5-7. Sham/Sham: mice initially infected with media as neonates and reinfected with media as young adults; A2/A2: mice initially infected with A2 as neonates and reinfected with A2 as young adults; rA2-19F/rA2-19F: mice initially infected with rA2-19F as neonates and reinfected with rA2-19F as young adults. The figure is a representative of 2–3 independent experiments. *:*p* < 0.05

As shown above, rA2-19F reinfection caused similar inflammation as A2 in the lung; and we further determined the types of these inflammatory cells by measuring BALF cellularity and pulmonary T cell profiles. Our data from the BALF (Fig. 8) show that reinfection recruited lymphocytes, neutrophils, and eosinophils into the lungs. Specifically, rA2-19F reinfection increased eosinophil recruitment into the airways as evidenced by increased percentages (12.23 ± 3.75 vs. 3.88 ± 1.06 %) and numbers (44,005 ± 19,218 vs. 7,531 ± 2,216 cells/ml) compared to A2 reinfection. No significant changes were observed in any other cell types, including monocytes/macrophages, lymphocytes, or neutrophils.

**Fig. 9:**
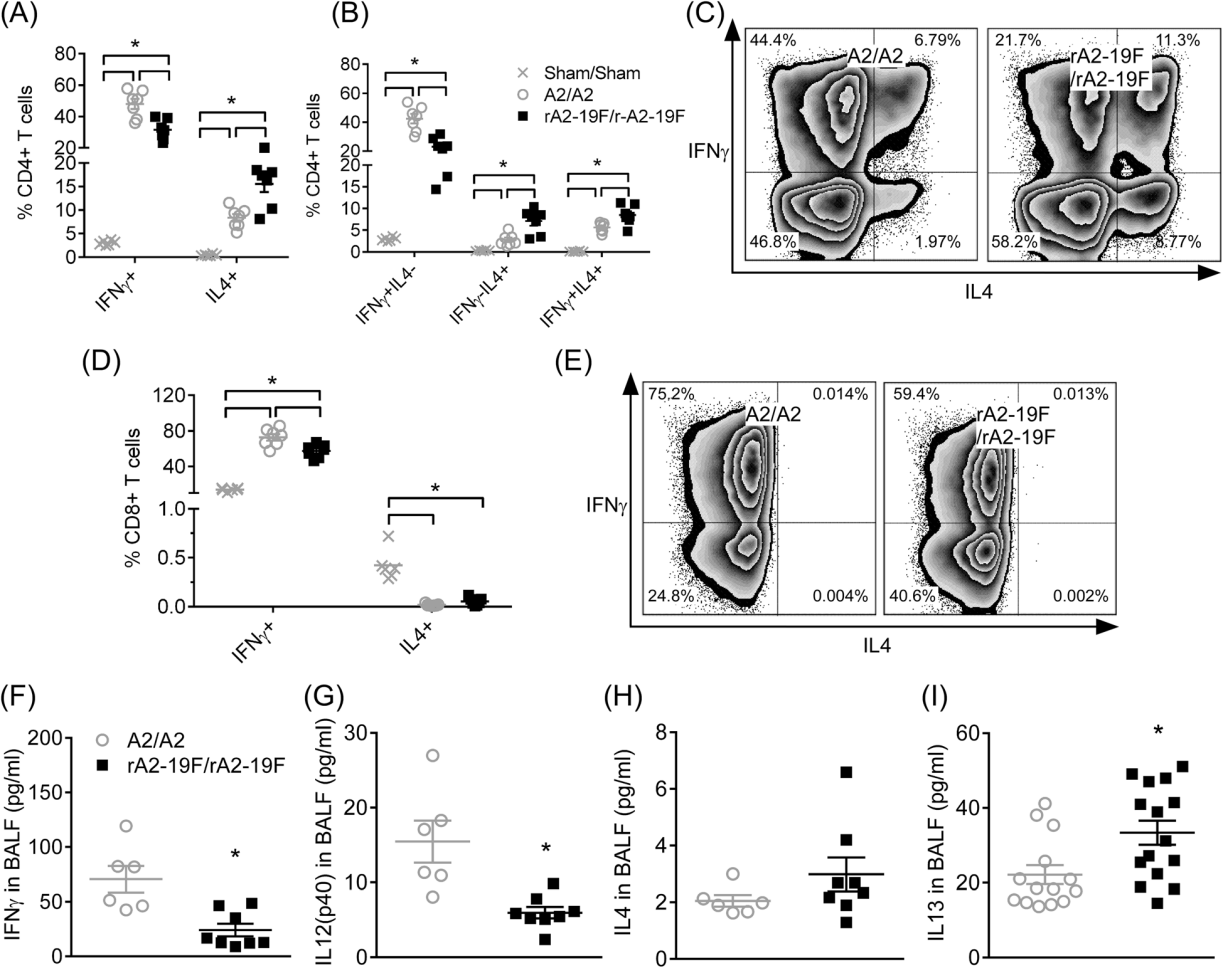
Exacerbated Th2 and reduced effector CD8^+^ T cells responses following reinfection with rA2-19F. Six days post reinfection, lung cells were isolated and stained with CD3, CD4, CD8, IFNγ, and IL4 for flow cytometry; and BALF were isolated and cytokines were measured. **a** CD4^+^ T cells expressing IFNγ or IL4. *N* = 5-7. **b** CD4^+^ T cell subsets (Th1: IFNγ + IL4-; Th2: IFNγ-IL4+; multi-functional Th: IFNγ + IL4+). **c** Representative flow plot of (**a**). **d** CD8^+^ T cell expressing IFNγ or IL4. *N* = 5-7. **e** Representative flow plot of (**c**). **f** IFNγ in the BALF. *N* = 5-8. **g** IL12(p40) in the BALF. *N* = 6-8. **h** IL4 in the BALF. *N* = 5-8. **i** IL13 in the BALF. *N* = 23–24. Sham/Sham: mice initially infected with media as neonates and reinfected with media as young adults; A2/A2: mice initially infected with A2 as neonates and reinfected with A2 as young adults; rA2-19F/rA2-19F: mice initially infected with rA2-19F as neonates and reinfected with rA2-19F as young adults. These figures are representative of 2 independent experiments. *:*p* < 0.05

While no significant changes were observed in the total CD3^+^ T cell population (data not shown), the types of these T cells in the lung differed between rA2-19F and A2 reinfected mice (Fig. 9a-e). Consistent with the observation at primary infection (Fig. 4), rA2-19F reinfection increased the frequency of IL4 expressing CD4^+^ T cells (15.54 ± 1.70 vs. 8.33 ± 0.82 %) and decreased the frequency of IFNγ expressing CD4^+^ T cells (31.46 ± 2.41 vs 48.08 ± 3.24 %). Specifically, frequencies of both Th2 cells (CD4 ^+^ IFNγ-IL4^+^) and multifunctional Th cells (CD4 ^+^ IFNγ ^+^ IL4^+^) were increased in rA2-19F vs. A2 reinfected mice. Furthermore, less effector CD8^+^ T cells (CD8 ^+^ IFNγ^+^) were observed in rA2-19F compared to A2 reinfected lungs (57.47 ± 2.87 vs. 72.85 ± 3.74 %).

Cytokine levels in the BALF were also consistent with above T cell observations (Fig. 9f-i). Less type I cytokines (IFNγ and IL12p40) and more type II cytokine (IL13) were detected in the BALF from mice reinfected with rA2-19F vs. A2. Specifically, IFNγ responses (24.11 ± 5.85 vs 70.55 ± 12.08 pg/ml) and IL12p40 (5.97 ± 0.76 vs. 15.45 ± 2.81 pg/ml) were reduced by almost 3 fold and IL13 (33.36 ± 3.23 vs. 22.15 ± 2.50 pg/ml) were increased by approximately 50 % in rA2-19F/rA2-19F vs. A2/A2 mice. No difference was observed in levels of IL4 between these two groups.

## Discussion

The current neonatal mouse model of infant RSV disease was established by our laboratories and other groups [9–11, 24], in which neonatal (<7d old) BALB/c mice are infected with the standard laboratory strain A2. Here, we established a new neonatal mouse model using the chimeric RSV (rA2-19F) in which the F protein from the A2 strain was replaced with the F protein from line 19 clinical isolate [17]. The infection of 5 days old BALB/c pups with rA2-19F induced less frequencies of Th1, less effector CD8^+^ T, and more Th2 cells in the lung compared to A2. The aberrant immune response was accompanied with severe lung injury characterized with significant interstitial and alveolar inflammation, airway mucus production and eosinophilia, and IL13 expression in lung homogenates. Upon reinfection, mice exhibited increased airway hyperreactivity, mucus hyperproduction, and eosinophilia with rA2-19F compared to A2. Consistently, mice reinfected with rA2-19F exhibited increased Th2 and decreased Th1 and effector CD8^+^ T cells in lungs with increased type 2 cytokine (IL13) and decreased type 1 cytokines (IFNγ and IL12p40) in airways compared to mice reinfected with A2.

Although it is out of the scope of this study, a possible mechanism that rA2-19F enhanced lung pathology and induced aberrant immune responses (increased Th2 and decreased Th1 and effector CD8^+^ T cells) compared to the A2 strain is that rA2-19F induces a more productive infection resulting in enhanced viral replication and higher pulmonary viral loads. Our data presented here in neonates agree with data presented by Dr. Moore’s group, which showed that higher pulmonary viral loads are observed in adult mice infected with rA2-19F compared to A2 or line 19 [17]. Further studies by this same group demonstrated that the F protein from line 19, and thus, in rA2-19F has a higher fusion activity compared to the F protein from A2 [32]. We believe the same to be true here; the F protein results in higher fusion activity in neonates and this results in elevated pulmonary viral loads.

The new neonatal mouse model presented here is an improved model due to the fact that primary infection with rA2-19F more closely mimics infection in human infection in infants compared to the A2 strain. In human infants, severe RSV infection causes substantial lung damage, including bronchiolitis and interstitial pneumonia and airway obstruction due to mucus production and epithelial cell sloughing [13]. Infection with rA2-19F induced significant interstitial inflammation and airway mucus production, whereas A2 infection induced only mild inflammation and mucus production in neonatal mice. Additionally, the immune responses induced by rA2-19F in neonatal mice are consistent with the responses induced by natural severe RSV infections in human infants. The innate immune response induced by natural RSV infection in infants is characterized with neutrophil influx in the airways [33]. Infection of neonatal mice with either virus strain (rA2-19F or A2) induced a significant influx of neutrophils into the airways compared to sham mice, while rA2-19F resulted in an enhanced, although not significant, recruitment compared to A2. The adaptive immune response during human infant RSV infection is generally Th2 biased [26–28]. Moreover, CD8^+^ T cells are thought to be protective in infants, since a lack of pulmonary CD8^+^ T cells is observed in fatal cases of RSV infection [34]. In this new model, primary infection of neonatal mice with rA2-19F induced a greater Th2 skewed response (higher frequency of Th2 cells, lower frequency of Th1 cells, and more IL13 expression) and further reductions in effector CD8^+^ T cell responses in the lungs compared to A2. The secondary immune responses to rA2-19F (i.e., reinfection) reflected the primary responses with an even stronger Th2 bias and further reduction of effector CD8^+^ T cells. These immune responses correlated to elevated airway hyperreactivity and mucus hyperproduction.

Recently, a manuscript using line 19 in neonatal mice was published [25]. This model successfully demonstrated that neonates infected with line 19 exhibit delayed and immature macrophage responses in the lung compared to adults and suggested that this deficiency in macrophage responses may explain RSV pathogenesis in infants. Similar to our data with A2 or rA2-19F compared to sham pups, Empey and colleagues observed a non-significant decrease in the percentage of BALF macrophages between mock pups and line 19 pups. Since we did not subphenotype the BALF macrophages, it is unclear, but highly likely that as with line 19 in neonatal mice, rA2-19F infection of neonatal mice results in an immature, delayed macrophage response. However, it is important to note that in our hands, pulmonary viral load following neonatal infection with rA2-19F was greater than that with A2 or line 19 and that A2 infection resulted in similar viral loads as line 19. Disease severity in humans has been positively associated with viral load. Also similar to human disease, neonatal infection with rA2-19F significantly increased mucus production in the airways, airways disease as evidenced by pathology, and airways resistance compared to A2. This was accompanied by modest increases in lymphocytes and eosinophils during primary infection.

As with all models, limitations exist. Most animal models of RSV, except chimpanzees, suffer from the fact that they are only semi-susceptible to human RSV infection. Although the infection of neonatal mice with rA2-19F resulted in higher pulmonary viral loads compared to A2, the peak pulmonary viral load at 4 dpi was still significantly lower than the inoculum suggesting that rA2-19F infection is still not optimal in mouse lung cells. This may be due to the infection route. For example, the mice are infected by inhaling a bolus dose of RSV intranasally, while human infants are typically infected by inhalation or aerosolized virus. In the mouse, our data demonstrate that approximately 70 % of the intranasally inhaled volume is directly delivered into the lung; however, the location where this distributes in the lung may not reflect that of a natural infection; and the resulting disease progression may be different.

## Conclusions

In summary, we present a new neonatal mouse model of severe RSV infection in infants. In this model, rA2-19F infection in neonatal mice induced enhanced lung pathology (interstitial pneumonia and mucus hyperproduction), exaggerated Th2 bias, and reduced effector CD8^+^ T cells during both primary and secondary infections compared to the standard laboratory strain A2. In addition, rA2-19F resulted in a higher viral load in the neonatal lung compared to A2 during primary infection. These data suggest that rA2-19F infection in neonatal mice is a better mouse model for RSV infection in human infants.

## Additional file

Additional file 1: Figure S1. Gating strategy for T cell subsets analysis. Live, CD3^+^ cells were first gated, then single, live, CD3^+^ cells in lymphocyte gate were selected. These CD3^+^ T cells were divided into CD4^+^ and CD8^+^ T cells subsets. Each subset were further classified into Th1 (CD4^+^ IFNγ ^+^ IL4-), Th2 (CD4^+^ IFNγ^-^IL4^+^), multi-functional Th (CD4^+^ IFNγ ^+^ IL4^+^) cells, Tc1 (CD8^+^ IFNγ^+^) and Tc2 (CD8 ^+^ IL4^+^) cells.

## Abbreviations

RSV: Respiratory syncytial virus
BALF: Bronchoalveolar lavage fluid
rA2-19F: The chimeric virus in which the F protein from A2 strain was replaced with the F protein from the line 19 strain.
Th1: T helper type 1
Th2: T helper type 2
TCID_50_: Tissue culture infectious dose
PMA: Phorbol-12-myristate-13-acetate
H&E: Hematoxylin and eosin
PAS: Periodic acid-Schiff
SEM: Standard errors
dpi: Days post-infection
