# Supplementary material for: Building a better neonatal mouse model to understand infant respiratory syncytial virus disease
Source: Respir Res. 2015 Aug 1;16(1):91. doi: 10.1186/s12931-015-0244-0 (PMC4531813; doi:10.1186/s12931-015-0244-0)
Supplement: Additional file 1: Figure S1. — Gating strategy for T cell subsets analysis. Live, CD3+ cells were first gated, then single, live, CD3+ cells in lymphocyte gate were selected. These CD3+ T cells were divided into CD4+ and CD8+ T cells subsets. Each subset were further classified into Th1 (CD4+ IFNγ + IL4-), Th2 (CD4+ IFNγ-IL4+), multi-functional Th (CD4+ IFNγ + IL4+) cells, Tc1 (CD8+ IFNγ+) and Tc2 (CD8 + IL4+) cells. [file 12931_2015_244_MOESM1_ESM.pdf]

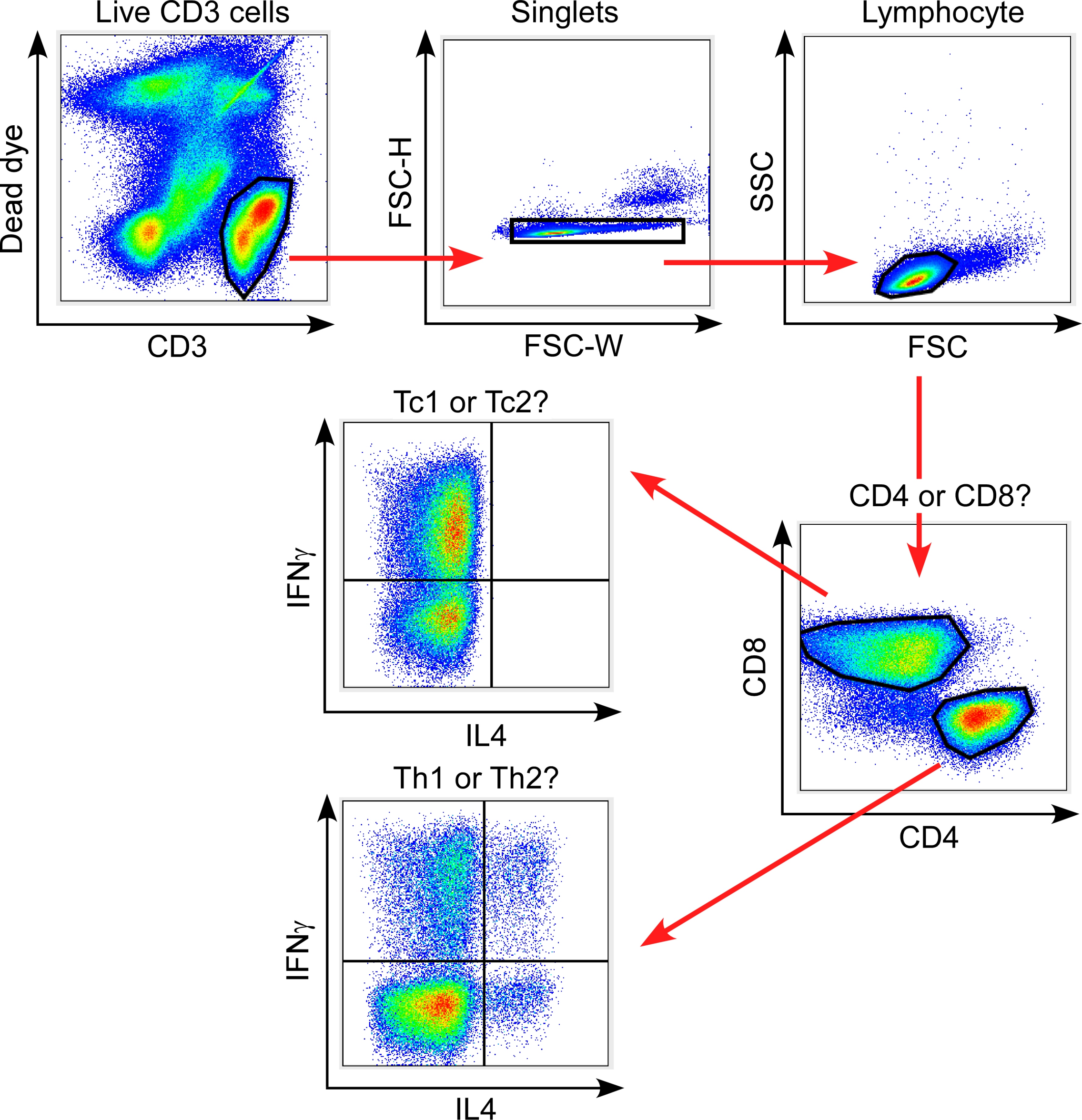

Live CD3 cells
Dead dye
CD3
Singlets
FSC-H
FSC-W
Lymphocyte
SSC
FSC
CD4 or CD8?
CD8
CD4
Tc1 or Tc2?
IFNγ
IL4
Th1 or Th2?
IFNγ
IL4

Supplemental Figure 1. Gating strategy for T cell subsets analysis. Live, $CD3^+$ cells were first gated, then single, live, $CD3^+$ cells in lymphocyte gate were selected. These $CD3^+$ T cells were divided into $CD4^+$ and $CD8^+$ T cells subsets. Each subset were further classified into Th1 ($CD4^+$ $IFN\gamma^+IL4^-$), Th2 ($CD4^+$ $IFN\gamma^-IL4^+$), multi-functional Th ($CD4^+$ $IFN\gamma^+IL4^+$) cells, Tc1 ($CD8^+$ $IFN\gamma^+$) and Tc2 ($CD8^+IL4^+$) cells.
